# Urine the Dark: A Sizzling Case of Delayed Thermal Ureteral Injury.

**DOI:** 10.51894/001c.144633

**Published:** 2025-09-30

**Authors:** Erica Redmann

**Affiliations:** 1 Department of Urology U of M Health Sparrow

## 117

### INTRODUCTION

Iatrogenic ureteral injuries are a rare but serious complication of pelvic surgery. Up to 82% of these injuries are due to gynecologic procedures but can also occur in colorectal and vascular surgeries. These complications can lead to significant cost, morbidity, and patient distress. Therefore, it is important to minimize their occurrence and raise awareness regarding how to avoid them.

### CASE DESCRIPTION

This is a case of a 54 year old female who underwent a laparoscopic converted to open total abdominal hysterectomy for fibroids and menorrhagia. She presented ten days later with worsening right flank pain and was found to have significant right hydronephrosis as well as delayed contrast drainage concerning for obstruction. There were no signs of contrast extravasation on her imaging, and there was a concern for complete transection or ligation of the right ureter. The patient was taken to the OR for further investigation. A right retrograde pyelogram demonstrated obvious contrast extravasation at the distal ureter. A double J stent was placed to allow for diversion of urine and adequate drainage. The patient will have the stent in place for 6 weeks and is scheduled for return to the OR for repeat retrograde pyelogram.

### CONCLUSIONS

This case highlights the delayed way in which ureteral injury can often present. Ureteral injuries typically have the best prognosis when identified immediately. However, thermal injury presents a unique challenge in that it reveals itself in a delayed fashion almost by definition. Therefore, to minimize their occurrence, it is vital to be aware of the risk factors for thermal iatrogenic ureteral injury and have a high suspicion in these cases.

